# Mathematical modeling supports the presence of neutrophil depriming in vivo

**DOI:** 10.1002/phy2.241

**Published:** 2014-03-20

**Authors:** Charlotte Summers, Edwin R. Chilvers, A Michael Peters

**Affiliations:** ^1^ Department of Medicine University of Cambridge School of Clinical Medicine Addenbrookes' and Papworth Hospitals Cambridge UK; ^2^ Brighton and Sussex Medical School Brighton UK

**Keywords:** Depriming, inflammation, neutrophil, priming

## Abstract

Following migration into the intestinal mucosa in inflammatory bowel disease (IBD), neutrophils enter the intestinal lumen and are excreted. This provides a basis for quantification of disease activity by measuring excreted label following injection of In‐111‐labeled neutrophils. In severe pan‐colitis, 50% of the injected In‐111 is typically recovered in the feces, indicating that 50% of neutrophil turnover is via fecal excretion. Neutrophils have an intravascular lifespan of ~10 h and a distribution volume of ~10 L, so total body neutrophil turnover is 10.*N*/10 cells/h, where *N* is the peripheral blood neutrophil count (cells/L). Neutrophil loss via the colon in a patient with 50% fecal In‐111 loss is therefore *N*/120 cells/min. Pan‐colonic mucosal‐blood flow in pan‐colitis is 200 mL/min, which would deliver *N*/5 neutrophils to the colon per min. Therefore, 5/120, or 4%, of incoming neutrophils undergo migration into inflamed bowel. If the 96% of nonmigrating cells exit in a primed state, then at steady state >90% of circulating neutrophils would be primed if no depriming took place. As the highest level of priming seen in IBD is ~40%, this indicates that depriming within the circulation must take place. Using the above values in the steady state equation relating priming rate to depriming rate plus primed‐cell destruction rate gives a mean depriming time of 35 min. We conclude that a very small proportion of neutrophils entering a site of inflammation migrate and that in vivo depriming must take place to limit the numbers of primed neutrophils in the circulation.

## Abstract 2

e00241

Following migration into the intestinal mucosa in inflammatory bowel disease (IBD), neutrophils enter the intestinal lumen and are excreted. This provides a basis for quantification of disease activity by measuring excreted label following injection of In‐111‐labeled neutrophils. In severe pan‐colitis, 50% of the injected In‐111 is typically recovered in the feces, indicating that 50% of neutrophil turnover is via fecal excretion. Neutrophils have an intravascular lifespan of ~10 h and a distribution volume of ~10 L, so total body neutrophil turnover is 10.N/10 cells/h, where N is the peripheral blood neutrophil count (cells/litre). Neutrophil loss via the colon in a patient with 50% fecal In‐111 loss is therefore N/120 cells/min. Pan‐colonic mucosal blood flow in pan‐colitis is 200 mL/min, which would deliver N/5 neutrophils to the colon per min. Therefore, 5/120, or 4%, of incoming neutrophils undergo migration into inflamed bowel. If the 96% of non‐migrating cells exit in a primed state, then at steady state >90% of circulating neutrophils would be primed if no de‐priming took place. As the highest level of priming seen in IBD is ~40%, this indicates that de‐priming within the circulation must take place. Using the above values in the steady state equation relating priming rate to de‐priming rate plus primed cell destruction rate gives a mean de‐priming time of 35 min. We conclude that a very small proportion of neutrophils entering a site of inflammation migrate and that in vivo de‐priming must take place to limit the numbers of primed neutrophils in the circulation.

## Introduction

The molecular mechanisms underpinning the migration of neutrophils from the blood into an inflammatory focus have been extensively investigated, and include vascular activation, neutrophil priming, neutrophil activation, chemotaxis, and trans‐endothelial migration (Ley et al. 2007). Against this background, however, the proportion of neutrophils that enter an inflammatory focus from the afferent blood supply is unknown. Neutrophil trafficking in inflammatory bowel disease (IBD) provides an opportunity to address this issue because of previous work using In‐111‐labeled neutrophils for imaging IBD and quantifying disease activity from the percentage of administered activity that can be recovered in the feces, or lost from the body (as determined by whole body counting). Moreover, it is known from the imaging aspects of this work that following migration into inflamed bowel, the vast majority of neutrophils gain access to the intestinal lumen and are excreted in the feces (Saverymuttu et al. 1982). In‐111 proves an almost ideal radionuclide for studying quantification of disease activity in IBD due to its half‐life, stability of intracellular binding (both in the neutrophil and other cells, such as reticulo‐endothelial macrophages, that subsequently ingest labeled neutrophils (Peters et al. 1982)), and the absence of downstream intestinal reabsorption of the label (as shown in gastro‐intestinal transit studies using orally administered In‐111) (Notghi 2006).

Disease activity in IBD was first quantified using In‐111‐labeled neutrophils by measuring In‐111 excretion in a 4‐day complete fecal collection following injection of the labeled cells (Saverymuttu et al. 1983, 1985a). The percentage of administered In‐111 excreted in the feces in normal subjects was essentially zero, but ranged from 10% in mild disease to >50% in severe and extensive disease. Typical values in severe disease are 40–50%. The evidence that all migrating cells access bowel lumen is based on imaging, which shows that following the appearance of substantial intestinal activity within the first few hours after injection, the label then moves distally over the subsequent hours and completely disappears from bowel over about 4–5 days (Saverymuttu et al. 1982, 1983, 1985a, 1986; Carpani de Kaski et al. 1992). In‐111‐labeled neutrophils migrating in severe bronchiectasis and swallowed in sputum also clear from the lungs and bowel within 5 days of injection (Currie et al. 1987).

## Theory

Neutrophils are distributed throughout the body between two pools: the circulating pool (= blood volume = 5 L) and the marginating pool, which between them make up the total blood neutrophil pool (Athens et al. 1961). It has been shown from imaging that the marginating pool is located predominantly within the liver, spleen, and bone marrow (Saverymuttu et al. 1985b). Neutrophils distribute about equally between the two pools so that the total blood neutrophil pool is equivalent to 10 L. Taking blood neutrophil count as *N* cells/L, the total intravascular population is therefore 10.*N*.

Neutrophils, radiolabeled with P‐32‐diisopropyl fluorophosphate (DFP‐32) or In‐111 are cleared from blood exponentially with a half‐time of ~7 h (Farahi et al. 2012; Athens et al. 1961; Saverymuttu et al. 1985b). This corresponds to a mean intravascular lifespan of 10 h, so intravascular whole body neutrophil turnover can be taken to be 10.*N*/10 cells/h = 10.*N*/600 cells/min = *N*/60 cells/min.

In severe pan‐colitis with 50% of the administered In‐111‐label lost into bowel lumen, neutrophil loss would be *N*/120 cells/min.

Colonic perfusion has been measured in several experimental settings, including nonanesthetized dogs, and is variable. Values for total flow range from 35 to 133 mL/min/100 g (Shoor et al. 1979; Bond et al. 1980; Ulrich‐Baker et al. 1986). Mucosal perfusion represents about 70% of total flow (Shoor et al. 1979). Measurements exclusively of mucosal flow using the microsphere technique are consistent with this, with values ranging from 20–130 mL/min/100 g (Bond et al. 1980; Mortensen et al. 1991). Mucosal flow increases in the inflamed colon by about 50% (Petersson et al. 2007). The empty colon weighs about 500 g, so the best estimate for pan‐colonic mucosal flow in an inflamed colon is 200 mL/min, which represents approximately 20% of splanchnic blood flow.

Therefore, neutrophil delivery to an inflamed colon in a case of pan‐colitis would be 0.2.*N* cells/min. So the percentage of neutrophils arriving in the inflamed colon on each pass that undergo migration is shown by:

(1) $$\%\text{Migrating}=\frac{100\times (N/120)}{N/5}$$

that is only 4%. If the inflamed colon had a mucosal flow of 100 mL/min, the percentage of incoming neutrophils undergoing migration would still be <10%.

Neutrophil half‐life, based either on DFP‐32 or In‐111 labeling studies, has been shown not to be shortened in inflammation (Athens et al. 1965; Saverymuttu et al. 1985b), but if it was, say 3.5 h instead of 7 h, then from equation (1), it can be seen the percentage of cells migrating would be ~8%. A recent study has suggested neutrophil half‐life may be much longer (Pillay et al. 2012), in which case the percentage migrating would become even smaller. However, the relevant half‐life is that of the labeled cells.

If the large percentage (96% in the above example) of neutrophils that entered colonic venous blood in IBD were nevertheless all primed due to their passage through an inflamed and cytokine‐rich site, this might explain the high levels of primed neutrophils found in the peripheral blood of patients with severe inflammatory disease, including IBD (Ussov et al. 1996). Knowing the percentage of afferent neutrophils that migrate allows us to speculate on the extent of spontaneous neutrophil depriming elsewhere in the circulation, the occurrence of which is controversial, as follows.

At steady state, assuming 4% migration and that the half‐life of primed cells is, like unprimed cells, 7 h, then:

(2) neutrophil priming rate=neutrophil de‐primingrate+primed neutrophil destruction rate

Typically, 40% of circulating neutrophils are primed in severe inflammation (Ussov et al. 1996), in which case we can say there are 6 units of unprimed cells and 4 units of primed cells per liter of circulating blood, giving totals of 30 units and 20 units, respectively. These totals will be the same in the marginating pool, giving 40 units of primed cells in the total blood neutrophil pool; therefore

(3) 0.2×6×0.96=40/t+40/600cells/min

where *t* is the mean time to deprime in vivo.

So *t *=* *37 min.

Lower levels of circulating primed neutrophils would, from equation (3), indicate faster rates of depriming; e.g. *t* would be 13 min for 20% priming.

If there was no in vivo depriming then, in the above scenario, the percentage of circulating primed cells would (from eq. (3)) be predicted to be >90%. Such high values are not encountered. In vivo depriming must therefore take place.

## Discussion

This theoretical treatment suggests that the proportion of neutrophils entering a site of inflammation that undergo extravascular migration is surprisingly small (far <10%). Such a small proportion provides the basis for the presence in peripheral blood of primed neutrophils in inflammatory disorders, including IBD, systemic vasculitis, systemic sepsis, graft‐versus‐host disease, severe alcoholic hepatitis and ARDS, if priming results, as assumed here, from the exposure of neutrophils to pro‐inflammatory cytokines as they pass through a site of inflammation. Arterio‐venous gradients for neutrophil priming and neutrophil count have not, to our knowledge, been directly measured across a site of inflammation.

Kitchen et al. (1996) demonstrated for the first time spontaneous depriming of neutrophils in vitro, following treatment with platelet activating factor. The halftime for depriming was about 30 min, corresponding to a mean depriming time of about 40 min, similar to *t* in the above calculations. Some priming mediators, such as TNF‐alpha and GM‐CSF, resulted in a far more prolonged priming response, so the rate of in vivo depriming presumably depends on which mediators neutrophils encounter as they pass through the inflammatory focus. We have assumed in equation (3) that all circulating primed neutrophils (*N*_p_) are reversibly primed. Depriming of reversibly primed cells would be faster if *N*_p_ consisted of both reversibly and irreversibly primed neutrophils.

Primed neutrophils change shape and become less deformable with the result that their transit time through the lungs is prolonged (Downey et al. 1990). Early work proposed that neutrophils pool physiologically in the lungs such that up to 90% of the total blood neutrophil pool resides within the pulmonary vasculature (Doerschuk et al. 1987). Following introduction of radiolabeled neutrophils for imaging inflammation, however, it became apparent that such a large pool was not compatible with routine imaging findings (where pulmonary uptake is minimal) and that the lung pulmonary pool only becomes expanded in patients with severe systemic inflammation, and then in proportion to the circulating fraction of primed cells (Ussov et al. 1996). It has recently been suggested that a prolonged pulmonary vascular transit time allows neutrophils to deprime before being released back into the systemic circulation in an unprimed state (Singh et al. 2012). Summers et al. (2009) injected In‐111‐labeled neutrophils primed ex vivo with GM‐CSF and found 100% retention in the lungs followed by a delayed release back into the circulation with a 40‐min halftime.

We have had to make several assumptions, as follows.

1. *Colonic mucosal blood flow is 200 mL/min*. This is perhaps the most contentious assumption. However, even assuming a blood flow of only 50 mL/min, which seems unfeasibly low, the fraction of neutrophils migrating would still be well under 20%.
2. *Mean neutrophil intravascular lifespan is 10 h, and not reduced in inflammation*. The relevant lifespan is that of the labeled cells, which for In‐111 has been demonstrated not to be reduced in inflammatory disease (Saverymuttu et al. 1985b).
3. *There is no difference between intravascular lifespans of primed and unprimed neutrophils*. Ex vivo, apoptosis is delayed in primed neutrophils (Murray et al. 1997). However, in vivo, neutrophils disappear from blood exponentially (Athens et al. 1961, 1965; Saverymuttu et al. 1985b); that is they are destroyed at random rather than any factor related to senescence. In any event, if the lifespan of primed cells was prolonged, it can be seen from rearranging equation (2) that we would have underestimated the spontaneous depriming rate.
4. *All primed cells have the potential to deprime*. This may not be true depending on the priming stimulus. In equation (3), *t* would therefore include values of infinity for irreversibly primed cells, and the de‐priming rate of reversibly primed cells would therefore be underestimated.
5. *After In‐111‐labeling, neutrophils retain the capacity to migrate normally*. This is very difficult to prove beyond doubt. However, neutrophils labeled using optimal separation techniques display a 40‐min intravascular recovery – 40–55% – (Ruparelia et al. 2011) close to the maximum achievable on the basis of what we know of normal neutrophil distribution between marginating and circulating pools (Athens et al. 1961, 1965; Saverymuttu et al. 1985b).
6. *All neutrophils exiting a site of inflammation are primed*. This assumption could be validated by microsampling of venular blood draining a site of inflammation, but to our knowledge this has not been done. If neutrophils in a capillary axial stream escaped priming, it is possible that only 50% of incoming cells would become primed. This would still result in a circulating *N*_p_ percentage of 86%, much higher than the highest priming percentages seen in severe IBD, which are about 40%.
7. *Transit time of primed neutrophils in the marginating pool is not prolonged*. If neutrophil transit time in the marginated pool was prolonged then the total blood neutrophil distribution volume would be greater. This would impact on equations (1) and (3). Assuming 40% neutrophils were primed and that the transit time in the marginated pool of primed neutrophils was twice that of unprimed neutrophils, then the effective volume of the marginated pool would be 7 L instead of 5, giving a total effective volume of 12 L. From equation (1), it can then be seen that the percentage of neutrophils undergoing migration would be 5% instead of 4%. In equation (3), there would be 40 units of primed neutrophils in the marginating pool instead of 20, giving a total of 60 units in the total blood neutrophil pool instead of 40. This would then give a mean time to deprime of 58 min instead of 37 min.

In conclusion, we suggest first that ≪10% of neutrophils delivered to a site of inflammation undergo migration, leaving >90% to continue to circulate. Second, we further suggest that these cells re‐enter the circulation in a primed state and thereafter undergo spontaneous in vivo depriming with a halftime of about 40 min, a prediction which fits well with in vitro experiments that examine the rate of spontaneous depriming in vitro.

## Conflict of Interest

None declared.
